# Effect of 6-Month Feeding with a Diet Enriched in EPA + DHA from Fish Meat on the Blood Metabolomic Profile of Dogs with Myxomatous Mitral Valve Disease

**DOI:** 10.3390/ani11123360

**Published:** 2021-11-24

**Authors:** Robert Pasławski, Agnieszka Kurosad, Adam Ząbek, Urszula Pasławska, Agnieszka Noszczyk-Nowak, Marcin Michałek, Piotr Młynarz

**Affiliations:** 1 Institute of Veterinary Medicine, Nicolaus Copernicus University, 87-100 Toruń, Poland; urszula.paslawska@umk.pl; 2 Vet Planet Sp. z o.o. ul. Brukowa 36/2, 05-092 Łomianki, Poland; dietwet@gmail.com; 3 Department of Bioorganic Chemistry, Faculty of Chemistry, Wroclaw University of Science and Technology, ul. Wybrzeże Wyspiańskiego 27, 50-370 Wrocław, Poland; adam.zabek@port.org.pl (A.Z.); piotr.mlynarz@pwr.edu.pl (P.M.); 4 PORT Polish Center for Technology Development, ul. Stabłowicka 147, 54-066 Wrocław, Poland; 5 Department of Internal Diseases with Clinic for Horses, Dogs and Cats, Faculty of Veterinary Medicine, Wroclaw University of Environmental and Life Sciences, ul. Norwida 31, 50-375 Wrocław, Poland; agnieszka.noszczyk-nowak@upwr.edu.pl (A.N.-N.); marcin.michalek@upwr.edu.pl (M.M.)

**Keywords:** metabolites, fatty acid, canine

## Abstract

**Simple Summary:**

Animal nutrition plays an important role in the therapy of many diseases, including heart failure. The aim of the research was to assess whether 6 months of feeding diet enriched in unsaturated fatty acids (from fish meat) in dogs suffering from heart failure due to mitral degeneration impacts the dogs’ metabolic profile and clinical status. Twenty small breed dogs in early stages of heart failure were randomly divided into two groups. One group receiving a standard diet, the second one a diet enriched in fish meat. All dogs continued to receive appropriate cardiac therapy throughout the study. Control examinations were performed at the start of the study, after 3 and 6 months of appropriate feeding. The results showed no differences in clinical, cardiological, haematological and biochemical parameters between the two study groups. The metabolomic changes was more pronounced with time. After 6 months of feeding the diete enriched in fish meat, there was a favorable reduction in glycerophosphocholine and xanthine levels, but an adverse increase in lactate and furvan and a decrease in alanine was not stopped.

**Abstract:**

Animal nutrition plays an important role in the therapy of many diseases, including heart failure. The aim was to assess whether 6 months of feeding an AEP + ADH enriched diet (from fish meat) in dogs suffering from heart failure due to mitral degeneration impacts the dogs’ metabolic profile and clinical status. Twenty small breed dogs were included: 50% were in stage B2 of MMVD and 50%, in stage C according to ACVIM. Dogs were randomly divided into two groups. One group receiving a standard diet, the second one a diet enriched with EPA + DHA (from fish meat). All dogs continued to receive appropriate therapy throughout the study. Control examinations were performed at the start of the study, after 3 and 6 months of appropriate feeding. Examinations included ECG, ECHO, blood hemathology and biochemistry, morphometric measurements, body fat index and subcutaneous fat tissue thickness. Serum samples were analyzed with a high-performance liquid chromatography system. Data were analyzed using the Progenesis QI (PQI, Non-linear Dynamics). The results showed no differences in clinical, cardiological, haematological and biochemical parameters between the two study groups. An effect on the metabolomic profile following a continued diet enriched in DHA + EPA (from fish meat) was more pronounced with time. After 6 months of feeding the diete enriched with DHA + EPA (from fish meat), there was a favorable reduction in glycerophosphocholine and xanthine levels, but an adverse increase in lactate and furvan and a decrease in alanine was not stopped.

## 1. Introduction

Animal nutrition plays an important role in the treatment of many diseases. Pathological changes lead to specific disturbances in the use of nutrients, which may lead to certain metabolic disorders. The idea behind a therapeutic diet is to match the energy and nutrient requirements during a disease state in such a way as to compensate for these disorders [1].

It is known that cardiac failure activates compensatory mechanisms. One of the most important of these is the renin-angiotensin-aldosterone axis, which is responsible for an increase in blood pressure. The main mechanism involved in the development of hypertension is sodium retention associated with simultaneous potassium loss. Therefore, the recommendation for functional foods in a prescription diet for cardiopathy according to the European Union is te limitation of sodium content to a value below 2.6 g/kg of a complete diet with a moisture content of 12% for pets [2,3]. So far, this is the sole “cardiologic” requirement for an applied diet that has been recommended.

There are, however, several other important nutritional ingredients, e.g., the long-chain fatty acids: eicosapentaenoic acid (EPA, 20:5 (*n*-3)) or docosahexaenoic acid (DHA 22:6 (*n*-3)), which can significantly support prescription diet for cardiopathy [4,5]. The reduced concentration of fatty acids (FA) in the blood serum increased after eating a fish-meat diet. Such a diet increases the concentration of fatty acids in blood serum, cells and tissues in humans and dogs with heart disease [5,6]. Metabolomic studies reflect the species-specific physiological and biochemical processes occurring during the development of heart failure and responses to nutrition change. Currently, two types of metabolomics analyses are performed: metabolomic fingerprinting and targeted metabolomics analysis. The evaluation of metabolite levels is a very sensitive tool since the current methodology allows for the detection of very small differences in their concentrations. Protein metabolites or simply amino acids have been found in many studies searching for biomarkers of specific diseases. Quite recently, using metabolomic methodologies, it was possible to identify metabolic inflexibility to food intake and lower carnitine concentrations in overweight Labrador Retrievers [7,8]. Up-to-date published papers revealed many different factors: feeding methods, nutritional habits of owners, food preparation methods, etc. influencing animal plasma or urine metabolomic response. One metabolomics study with serum transcriptomic analyzes of serum identified cellular and metabolic pathways that play a role in myxomatous mitral valve disease (MMVD). The study results identified 41 known and 13 unknown serum metabolites that were significantly different between healthy and MMVD dogs, representing alterations in fatty acid and glucose energy metabolism, oxidative stress, and other pathways [9]. There is very limited information on the influence of supplementation of unsaturated fatty acids in dogs with MMVD and only one of them establishes the impact of diet modification on the metabolomic profile in MMVD dogs [10].

The aim of this study was to assess whether a 6-month administration of a diet enriched with EPA + DHA from fish meat in dogs suffering from heart failure due to MMVD improves their metabolic profile and clinical status.

## 2. Materials and Methods

### 2.1. Ethical Statement

During the meeting of the Local Ethics Committee, in Wroclaw of 23 September 2021 and 27 October 2021 the members unanimously decided that the procedures followed are standard procedures with treatment used in veterinary patients (including blood test in order to verification the treatment course in 3 and 6 months of treatment). In the opinion of members Local Ethics Committe, the research did not include experimental procedures in view of the Act of 15 January 2015 on the protection of animals used for scientific or educational purposes and is not subject to assessment Local Ethics Committee.

### 2.2. Animals

Dogs were selected for this prospective study based on a clinical history, physical examination and additional cardiac examination. Inclusion criteria included chronic cardiac disease (in the stable phase) due to degenerative mitral changes. Based on echocardiographic results, all dogs included in the study had thickened mitral valve leaflets, significant mitral regurgitation and a resulting enlarged left atrium (LA/Ao > 1.5). Dogs with MMVD stages B2 or C2 according to ACVIM classification being 50% of them classified as B2 and 50% as C2. [11]. Dogs were qualified for the study with the consent of the owners. The owners consented to only feed dogs with the prescribed diet and report for the designated control examinations. An additional necessary condition was the acceptance of the prescription diet by the dog. For this, the amount of the prescribed diet was gradually increased over the 2 weeks prior to the start of the study. If, after 14 days of adaptation, the dog was willing to follow the prescribed diet and had no side effects, that day was considered as the beginning of the study. Dogs had unlimited access to water. The exclusion criteria were: periods of growth (under 15 months of age), pregnancy, lactation, convalescence, arrhythmia, the presence of diseases that could significantly affect the metabolism e.g., cancer, central nervous system diseases, endocrine and immunological dysfunction, the presence of acute respiratory failure or other acute inflammatory process, or taking non-cardiac drugs. Only small-size breed dogs were included in the study because earlier studies have documented the presence of metabolic differences compared to medium and giant-sized breeds. [5,12].

During the study, the dogs continued their current pharmacological treatment. Pharmacological protocol for stage B2 included: benazepril hydrochloride (0.25 mg/kg), and for stage C2 benazepril hydrochloride (0.25 mg/kg), pimobendan (0.2–0.6 mg/kg), and diuretic (spironolactone (2 mg/kg) and furosemide (1–3 mg/kg) or torasemide (0.1–0.6 mg/kg)) in doses recommended by the manufacturer. Furosemide and torasemide were used in relation to respiratory distress. Twenty-eight dogs were qualified for the study and were randomly assigned into one of two experimental groups: dogs fed a premium commercial diet for adult maintenance or a prescription diet for cardiopaties enriched with EPA + DHA (from fish meat) (Table 1).

Four dogs (eight dogs in total) were excluded from each group during the experiment. The reasons for exclusion were different (two owners did not come to the follow-up visit, four owners admitted that they provided additional food, two were excluded due to deterioration of blood tests—one dog was later diagnosed with leukemia, the other was diagnosed with liver neoplasia).

### 2.3. Diets Composition

Dogs from the diet group received a dry prescription diet enriched in EPA + DHA (from fish meat) (Table 2), while the second group received a dry maintenance diet (with lamb meat). Inevitably the addition of fish meat changed many nutrients in addition to omega 3 acids. Dogs in both groups received test diets in an amount appropriate to the daily energy requirement (DER) of mature dogs, i.e., over the age of 7, which is: 80–120 kcal ME/kg^0.75^; average: 95 kcal ME/kg^0.75^ (3). The study did not interfere with the regulation of body weight, therefore the basis for calculating the DER was the current and not the optimal body weight of dogs. The parameters of both products are given in Table 2.

### 2.4. Study Protocol

The experiment lasted 6 months. The tests were performed at three time points: Point 0—start of the study, 1st time point—after 3 months and 2nd time point—after 6 months of feeding the tested diets. After the third month, the examinations were performed for two reasons. First, to see if there were any facts during this period that could significantly affect the test result after 6 months, e.g., the appearance of another organ disease or the administration of a different food. In this case, the dog was excluded from the test group. Second, to assess whether longer DHA + EPA supplementation had a stronger effect than the shorter one.

#### 2.4.1. Hematological and Serum Biochemical Tests

A 12 h fasting period preceded blood collection from the saphenous vein. Hematological tests were performed immediately after taking 2 mL of blood into an EDTA tube. The tests were performed using IDEXX X LaserCyte (Tokyo, Japan)^®^ and Horiba ABC Animal Blood Counter (Montpellier, France)^®^. A complete blood count was made: WBC: white blood cells; RBC: red blood cell count; HGB: concentration of hemoglobin; HT: hematocrit; MCV: mean red cell volume; MCH: mean mass of hemoglobin in the red cell; MCHC: concentration of hemoglobin in the red cell; PLT—platelet (thrombocytes) count. Biochemical analyses were performed on the blood serum, after centrifugation, using the TermoScientific Konelab Prime 30ISE (Vantaa, Finland)^®^ biochemical analyzer in the analytical laboratory of the Department of Internal Diseases of Horses, Dogs and Cats. The concentration of sodium (Na), potassium (K), ionized calcium (Ca^2+^), magnesium (Mg), iron (Fe), glucose, urea, creatinine, total protein, albumin, aspartate aminotransferase (AST), alanine aminotransferase (ALT), C-reactive protein (CRP) were determined.

#### 2.4.2. Electrocardiographic (ECG) and Echocardiographic (ECHO) Examinations

**Figure 1:**
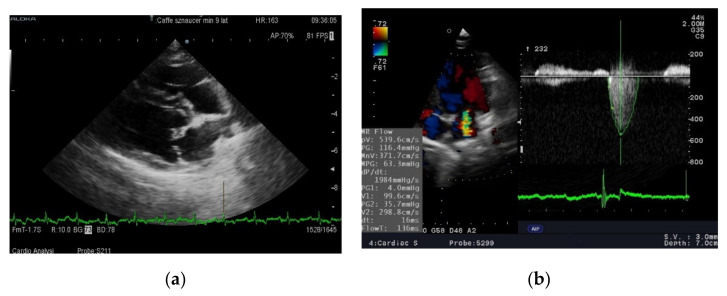
Echocardiographic examination of a dog with MMVD (**a**) 4th right intercostal space, long axis four chamber view. Tips of the mitral leaflet are thickened and deformed by degenerative processes—arrow. (**b**) On the left side of image is presented a multi-colored, turbulent jet of blood flowing back into the left atrium during systole. 5th left intercostal space, apical four-chamber view. On the right side—measurement of mitral regurgitant flow using continous Doppler method.

Standard ambulatory ECG examinations were performed using the digital BTL^®^ electrocardiograph in the standing position. A standard ECHO examination was performed using the Aloka Alpha 6 (Osaka, Japan)^®^ with a 5–7.5 MHz sector probe. The aortic diameter (Ao), left atrium diameter (LA), HR: heart rate; LA/Ao: left atrium to aorta diameter ratio; end-diastolic (RVIDd) internal diameter of the right ventricle, end-diastolic (LVIDd) and end-systolic (LVIDs) internal diameter of the left ventricle, thickness of the interventricular septum at end diastole (IVSd) and end systole (IVSs), and the end-diastolic (LVFWd) and end-systolic (LVFWs) thickness of the free wall of the left ventricle were measured. The shortening fraction (FS) of the left ventricle was calculated using the Teicholz formula (FS = [(LVIDd−LVIDs):LVIDd] × 100) [13]. Mitral regurgitation, maximal flow of mitral regurgitation (MR) was measured using continuous wave Doppler technique from the left parasternal two- or four-chamber view. All the dogs included in the study had degenerative malformations on the tips of the mitral leaflets and elevated left atrium/aorta ratios (stage B2 according to ACVIM classification) [11]. Dogs in stage C2 additionally had clinical signs of cardiac failure (exercise intolerance and occasionally cough). All these dogs have mitral regurgitation flow into the left atrium (visualized and measured using the Doppler technique) (Figure 1a,b). The experiment was double blinded: owners and cardiologist were blinded to diet received by dog.

#### 2.4.3. Morphometric Measurements, Body Fat Index, Subcutaneous Fat Tissue Thickness

At the beginning of the study and the feeding regiment, the dogs underwent morphometric measurements: pelvic circumference, hock to stifle distance, head length, measured from level of medial cantus equidistant between eyes to the external occipital protuberance, head circumference, measured between eyes and ears at widest part of head, front leg length, measured from proximal edge of central foot pad to the olecranon process with the carpus in a straightened position and hind leg length, measured from proximal edge of central foot pad to the dorsal tip of calcaneal process with the tarsus in a straightened position [14,15]. Morphometric measurements were done to assess the body fat tissue and subsequently fat index. The fat index (% of body fat tissue) was calculated according to the Burkholder method [14]. Additionally, subcutaneous fat tissue thickness was measured using ultrasonography (Hitachi-Aloka F37 (Tokyo, Japan) with a linear probe 7–10 MHz) over the scapula, parallel to abdominal linea alba and lateral to the lumbar vertebrae. None of the dogs in this study were cachectic or underweight.

#### 2.4.4. Metabolic Fingerprinting with Liquid Chromatography-Mass Spectrometry (LC-MS)

Samples were analyzed by a high-performance liquid chromatography (HPLC) system that consisted of a degasser, two binary pumps and Thermostatted Autosampler (Acquity UPLC I-Class, Waters, Ireland) connected to a waters Synapt G2-Si HDMS QuanTOF mass spectrometry detector. Serum samples (10 μL) were applied to a reversed-phase column (HSS T3 2.1 × 100 mm, 1.8 μm; Waters, Ireland) thermostatted at 40 °C. The system was operated at a flow rate of 0.3 mL/min with solvent A—water with 0.1% formic acid and solvent B—acetonitrile with 0.1% formic acid. The total analysis time lasted 10 min. The gradient started from 1% of B during the first 0.25 min, to 99% in 6.5 min. Next, the gradient was returned to starting conditions in 0.1 min, keeping the re-equilibration for 10 min. Data was collected in ESI positive (+) and negative (−) ion modes in separate runs on a QuanTOF operated in MS^E^ mode from 50 *m*/*z* to 600 *m*/*z* with a scan rate of 0.1 scan per second. Accurate mass measurements were obtained by means of an automated calibrant delivery system that continuously introduces a calibrating solution, which contains reference masses at *m*/*z* 120.0813 and *m*/*z* 556.2771 (Leucine-Enkephalin) in positive ion mode; and *m*/*z* 179.0821 and *m*/*z* 554.2615 in negative ion mode. The capillary voltage was set to 1500 V for positive and 3000 V for negative ionization modes; the drying gas flow rate was 800 L/h. Samples were analyzed in a randomized order in separate runs (first for positive and then for negative ion mode). At the beginning of each run, a batch of ten injections of a QC sample was used to condition the column.

#### 2.4.5. LC-MS Data Treatment

Raw data collected by the analytical instrumentation was analyzed by Progenesis QI (PQI, Non-linear Dynamics). Due to subtle retention time shifts, samples needed proper alignment to ensure the same metabolite was listed as the same feature within each sample analysis. Therefore, samples were multi-aligned using PQI software.

After successful alignment, PQI moved on to the peak picking stage. For this purpose, the absolute ion intensity method was chosen (groups of peaks with an absolute intensity level less than the given value were ignored). The limit for the background noise was set to 200 counts, and to find coeluting adducts of the same feature, the following adduct settings were applied: +H, +Na, +K, +ACN+H in positive ion mode and: −H, FA−H for negative ion mode. Dehydration neutral losses were also allowed. Parameters applied for the alignment were 1% for retention time correction and 10 ppm for correction of the mass. In further analyses, metabolic features with a CV% > 30% were used. Progenesis QI using the Progenesis MetaScope search engine and widely available databases has identified metabolic features. List of metabolites selected by VIP model: Ascorbalamic acid, Glycerophosphocholine, Kynurenic acid, L-Alanine, L-Asparagine, L-Glutamine, L-Lactic acid, L-Lysine, L-Tryptophan, Ornithine, Propylene glycol, Pyroglutamic acid, Pyruvic acid, Uric acid, Xanthine, Glycerophosphocholine.

#### 2.4.6. Statistical Analysis

The differences between groups were tested using a T student algorithm. The Anova and Bonferroni methods were used for within-group comparisons (Statistica 10). Multivariate data analysis was performed using the SIMCA software (v 14.0, Umetrics). Sample order in datasets was randomized. All variables were scaled to unit standard deviation. *p* value < 0.05 was significant. 

Both foods were well tolerated by dogs. Appetite was maintained in both groups and no additional clinical signs were found during routine clinical examinations.

## 3. Results

### 3.1. Blood Examination

The results of blood tests did not show differences in hematological (Table 3) and biochemical variables (Table 4) between the examined groups or between particular time points within the group.

### 3.2. Subcutaneous Fat Tissue Thickness

Measurements of the thickness of the subcutaneous fat showed no significant differences between the two groups, or between different time points (Table 5).

### 3.3. Electrocardiographic and Echocardiographic Examinations

The results of electro- and echocardiographic examinations did not show differences between the examined groups or between particular time points within the group (Table 6).

### 3.4. Metabolic Fingerprinting Data Analysis

In total, 56 metabolic traits in positive and negative ionization were identified based on experimental data, information published in the literature, Progenesis QI software (PQI, Non-linear Dynamics) as well as the METLIN Mass Spectral Database. Available online: https://sciencesolutions.wiley.com/wp-content/uploads/2020/06/Wiley_METLIN-Mass-Spectral-Database_978-1-119-37705-4.pdf (accessed on 23 November 2021) and Human Metabolome Database. Available online: https://academic.oup.com/nar/advance-article/doi/10.1093/nar/gkab1062/6431815 (accessed on 23 November 2021) Figure 2 shows separate data sets for groups of dogs at two time points, with 1A at 3 months and 1B at 6 months.

Based on the parameters of PLS-DA models, it is evident that a better separation between groups was obtained after 6 months from the initiation of the DHA + EPA rich prescription diet. At the same time, the VIP-PLS-DA analysis of selected variables shows which diet has the greatest impact on the distribution of samples at individual time points. The data is presented in Table 7 and Table 8.

Although the model parameters were not satisfactory and the ANOVA CV test result was negative, many individual metabolites affected group separation. It is noteworthy that the percentage differences between some metabolites are small, so those for which PD was greater than 10% were analyzed further. Elevated metabolites in dogs fed the EPA + DHA rich prescription diet after 3 months included pyroglutamate and propylene glycol, while the down-regulated metabolites included uric acid, xanthine, tryptophan, kynurenic acid, aspartate, glutamate and ornithine.

Up-regulated metabolites at the 6 month point of the EPA + DHA rich prescription diet included lactate, pyruvate and aspartate, while decreased values were noted in alanine, xanthine and glycerophosphocholine.

## 4. Discussion

Our research presents the first contribution on the impact of supplementation of DHA + EPA from fish meat on metabolism in dogs with MMVD stage B2 and C2. There are many reports that polyunsaturated omega-3 and omega-6 fatty acids have a very beneficial effect on the metabolic functions of the body. In both humans and animals receiving an enriched diet with essential fatty acid, especially omega-3 fatty acids, a reduced morbidity and lower mortality have been noted in people in the course of heart disease [16,17,18]. Omega-3 polyunsaturated fatty acids must be systematically supplied in the diet because they belong to exogenous compounds, which cannot be synthesize de novo in the mammalian body. The precursor of the omega-3 family is alpha-linolenic acid (18:3 omega-3), which by elongation and desaturation of the carbon hydrate chain is converted into a biologically more active EPA (20:5 omega-3) and DHA (22:6 omega-3). The metabolism of alpha-linolenic acid is subject to large individual fluctuations, but generally is inefficient because no more than 5% is converted to EPA and less than 1% to DHA [5]. Therefore, increasing tissue EPA and especially DHA levels via dietary supplementation would be more effective to the sick dogs. Depending on the source of origin, fatty acids differ in quantity and composition: fish and fish oils are rich in long chain *n*-3 FA, mostly EPA and DHA, whereas terrestrial plants only provide alpha linolenic acid [19,20]. Polyunsaturated fatty acid (PUFA) omega-3 and omega-6 metabolites have a significant, but different, effect on cellular biochemical processes, therefore the proper ratio between both acids should be retained in the diet. The optimal ratio of omega-6 PUFA to omega-3 PUFA in healthy dogs should be 5–10:1 [21]. When this ratio is inappropriate, the effect may be counterproductive. In a typical human “western diet” the ratio of omega-6 to omega-3 PUFA increases to 15–16.7/1 and can be an independent factor in the development of heart failure [21,22]. Several strategies are used to increase the amount of *n*-3 PUFAs in the bloodstream: (1) increased consumption of fish fats or their meat rich in *n*-3 PUFAs, (2) enrichment of food products with fish oil and alpha-oleic acid, (3) enrichment of PUFAs in meat of farmed animals by using a diet rich in *n*-3 PUFAs, (4) increasing the amount of *n*-3 PUFAs in oilseed crops by genetic engineering [23,24,25,26].

The first strategy increasing the intake of *n*-3 PUFAs (DHA + EPA) by increasing the amount of fish fat and meat was used. Despite the fact that dogs from both experimental groups accepted their diets, a decrease of body weight and fat index was observed in both groups. A decrease of subcutaneous fat was more visible after 3 months than at the end of the study, but the differences between groups were not significant. Owners considered the diet enriched in DHA + EPA (from fish meat) as tasty for their pets and it was very readily accepted by dogs (several owners noticed improvement of coat, and an increase of vitality). However, some animals lost their initial enthusiasm after a few weeks on the diet. We suspect that loss of body weight may be related to the categorical prohibition of giving dogs snacks during the course of the study and strictly limiting them to the tested diet. The average weight loss was more pronounced in the group of dogs receiving the standard diet, but the differences between groups was not significant.

Previous studies have shown that omega-3 PUFAs have many beneficial effects in human heart failure including inhibition of proinflammatory response in patients with increased concentration of TNF-alfa, IL-1 and IL-6 [27]. This effect is secondary to decreased activity of transcriptional factor NF-kappaB, which controls cytokine synthesis (and whose activity increases pathologically in heart failure) [28,29]. In the canine MMVD transcriptome there is a consistently increased expression of inflammatory genes; predominantly the expression of toll-like receptors and interleukins, which are involved in both the control of inflammation as well as other biological pathways in dogs [10] and people [30,31]. Earlier research revealed that the equilibration process of *n*-6 FA is slow and the beneficial anti-inflammatory effects of dietary *n*-3 FA supplementation in dogs takes a few weeks [32]. In our study, despite a 6 month feeding period, neither diet was found to affect leukocyte counts or CRP levels with values remaining within normal ranges throughout the observation period in both groups. It seems, therefore, that at the B2 and Cc stages of MMVD the activation of inflammatory processes is too low for the effect of anti-inflammatory diets to be seen. Studies documenting the increased activity of proinflammatory factors were performed in dogs asleep due to advanced heart disease. Neither electrocardiographic nor echocardiographic parameters revealed any changes. This finding is different from the results of Li et al. [9]. However, in this experiment, a statistically significant reduction in left atrium size was only observed in a group of previously untreated dogs. The reduction in left atrium size was correlated with a drop in blood pressure and was not significant in dogs that had previously received angiotensin converting enzyme inhibitor (ACE-I). In our study all dogs were treated, which may explain the difference in results. There was a clear (slightly below statistical significance) increase in left ventricular contractility as measured by the shortening and ejection fraction in dogs fed a diet rich in unsaturated fatty acids after 3 months. The difference disappeared after 6 months and the systolic function in both groups was almost identical. Routine diagnostic tests were not sensitive enough to assess whether feeding with a diet enriched in DHA + EPA inhibits or slows down pathological processes occurring in valves or heart muscle in dogs with MMVD. Therefore, it was decided to evaluate the effect of the studied diet on the metabolomic image.

To explain the metabolic changes that occurred during the study, a widely used methodology for serum metabolomic profiling using liquid chromatography coupled with mass spectroscopy was chosen. There are many examples in the literature of the use of metabolomics in the diagnosis of human cardiovascular disease caused by atherosclerosis [28], swine model of atherosclerosis [33,34], coronary heart disease [35,36,37] and hypertension [38]. This method has also been used successfully in studies on animals: rats with hypertension and myocardial infarction, pigs with myocardial infarct and dogs with MMVD [39,40,41].

In our dogs, the results of the analyses show that the effect of diet on metabolism increases over time. The differences after 6 months were significantly greater than after 3 months. Of the many metabolic characteristics determined by mass spectrometry, accurate assignment of names to compounds involved in the differentiation of both groups of animals was possible for only six metabolites. It should be noted that these are not the only variables-metabolites generating separation between observations, Table 6. Metabolites that increased after 6 months of feeding dogs with a diet rich in DHA + EPA included: lactate, pyruvate and aspartate, while reduced values were noted for alanine, xanthine and glycerophosphocholine. Although the diet enriched with an additional source of omega-3 DHA and EPA should have a positive effect on changes in metabolism, an increase in blood lactate is observed.

As it has been shown in very extensive studies devoted to DHA and EPA influence on Atlantic salmon fish, both omega-3 acids divergently induced gene expression associated with glucose metabolism. DHA is responsible for glycolytic pathways with the production of pyruvate and lactate. While EPA and EPA + DHA was positively associated with glycogen degradation to glucose, simultaneously EPA was inducing gluconeogenesis by specific gene glucose-6-phosphatase which release glucose. An increase in lactate and pyruvate may be primarily associated with accelerated glycolytic pathways of DHA (anaerobic glycolysis) [42]. An increase in lactate is usually associated with a decrease in glucose. However, in dogs fed a diet enriched with DHA + EPA (from fish meat), glucose levels were stable (and even increased statistically insignificantly compared to the first study), which can be associated with EPA. This indicates that despite increased glycolysis, the mechanisms that control blood glucose remain functional. A decrease in pH associated with increase in lactate helps to increase the use of fatty acids in all myocytes as an energy source via increase pyruvate dehydrogenase activity [43]. It has been proven in human studies with the use of isotope techniques that the increase in the metabolic rate of PUFA has a “sparing” effect on the use of intramuscular glycogen stores (promotes the reconstruction of glycogen reserves in muscles) and may also occur in conditions of no changes in blood glucose [44].

Increased asparagine levels in the group of dogs fed the DHA + EPA diet may indicate increased nitrogen transport from cells. Asparagine can be converted into aspartate, and this—as a glucogenic amino acid—can be transaminated into oxaloacetate (a key regulator of levels of Krebs cycle intermediates). This reaction is critical because it allows aspartate and asparagine stores to serve as absorbers for excess oxaloacetate produced from supplemented α-ketoglutarate precursors.

Dogs fed a diet enriched with DHA + EPA (from fish meat) had decreased levels of alanine. Alanine metabolism is tightly associated with gluconeogenesis, which can be directly influenced by tricarboxylic acid cycle via pyruvate [40]. The decrease in alanine levels correlates with increased amounts of lactate and pyruvate. This fact can confirm the assumption that despite using a diet rich in omega-3 fatty acids, heart disease increases glycolysis, at the expense of oxygen metabolism of fatty acids. A reduced level of glycerophosphocholine, a phospholipid precursor, is also noteworthy. Reports suggest that glycerophosphocholine metabolites may be closely related to the risk of heart disease [45]. Therefore, its lowered serum level may be the first positive clinical sigh of diet enriched DHA + EPA (from fish meat).

As documented in humans the second beneficial effect is decrease of xantine, which is an intermediate product in purine catabolism. Purines have an influence on receptors (in particular the different types of P2Y-receptors) in blood vessels and the heart, where they are involved in the progression of heart failure [46]. Increased levels of xantine were observed in patients with acute coronary syndrome and arteriosclerosis [47,48].

We suppose that such a negligible (contrary to literature data) beneficial effect of a diet rich in DHA + EPA was the result of including dogs in a relatively early stage of heart failure [4,5,6,16,24,28,29]. During early stages of heart disease, metabolic disturbances can be almost completely counterbalanced by compensatory mechanisms. It is worth noting that heart failure is dominant in dogs over 7 years of age, while better effects of increasing the concentration of (*n*-3) FA were observed in younger than older dogs [49]. Only one metabolic analysis of the serum of dogs with MMVD has been published showing an increase of 102 metabolites including arginine, α-aminobutyrate, citrulline, caprate, deoxycarnitine and sphingomyelin. The margarate and methyl palmitate levels decreased [10]. Our research confirms and complements this knowledge regarding the effects of unsaturated fats on the metabolism of dogs with MMVD, although the studies carried out have a number of limitations. First of all, there was a relatively small number of dogs in the groups, and secondly, the differences in the composition of the diet caused by the addition of fish meat. Moreover, we could not distinguish the effect of fatty acids from the effect of other nutrients in fish meat and it cannot be ruled out that the effects of diet enriched in DHA + EPA (from fish meat) in dogs with more advanced stages of heart failure may not be the same.

## 5. Conclusions

The results showed no differences in clinical, cardiological, haematological or biochemical parameters. The effect on the metabolomic profile following a diet enriched in DHA + EPA (from fish meat) was more pronounced the longer the dogs were fed the diet. After 6 months of feeding the diet enriched in DHA + EPA (from fish meat), there was a favorable reduction in glycerophosphocholine and xanthine levels, but the increase in lactate and furvan and the decrease in alanine were not stopped.

## Figures and Tables

**Figure 2:**
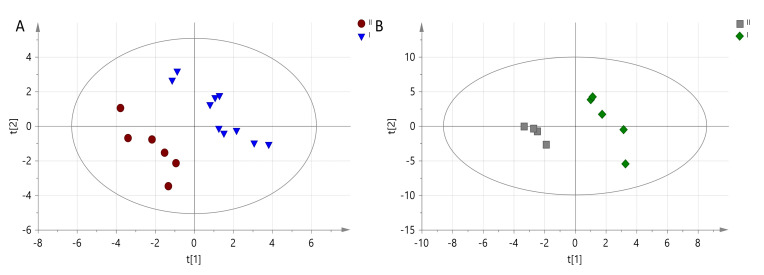
PLS-DA model obtained from LC-MS data for (**A**) 2nd time point and differences between Diet I (EPA + DHA rich prescription diet) marked and Diet II (maintenance diet) marked-R2X = 0.183, R2Y = 0.942, Q2 = N/A; (**B**) 3rd time point and differences between Diet I marked and Diet II marked-R2X = 0.334, R2Y = 0.981, Q2 = 0.342.

**Table 1:** Clinical data of enrolled dogs at the start of the research.

Parameter	EPA + DHA Rich Prescription Diet *n* = 10	Maintenance Diet *n* = 10	*p* Value
Age (years)	10.8 ± 2.5	9.5 ± 3.5	>0.1
Sex (female/male)	2/8	3/7	0.1
Body weight (kg)	8 ± 2.5	8.9 ± 4	>0.1
Body fat (%)	8–36	11–32	>0.1
Breed	CKCS, Miniature Schnauzer, 8 mixed breed	2 CKCS, Miniature Schnauzer, Shi-tzu, 6 mixed breed	
Number of dogs in ACVIM stage B2/Cc	4/6	5/5	>0.1

CKCS—Cavalier King Charles Spaniel.

**Table 2:** Composition of dry diets—analysis per 100 g. Test performed in Hamiton Poland Laboratory.

Nutrient	EPA + DHA Rich Prescription Diet	Maintenance Diet
Crude protein (g)	26	27
Crude fat (g)	19	16
Crude fiber (g)	1.7	2.5
Crude ash (g)	5	6.5
Water (g)	8.5	10
Carbohydrates (g)	39.8	38
Ca (g)	0.85	1.4
P (g)	0.75	1
*n*-3 (g)	2.5	0.176
*n*-6:*n*-3	1:1	10:1
DHA + EPA (g)	1.87	0.064
GLA (g)	0.09	not declared
Vit A (IU)	1818	1500
Vit D (IU)	92	150
Vit E (mg)	55	50
Na (g)	0.21	0.26
Taurine (mg)	250	not declared
Energy (kJ/100 g)	1816	1690
Per MJ:		
Protein (g/MJ)	14.3	16.0
Ca (g/MJ)	0.47	0.83
P (g/MJ)	0.41	0.59
Zn (mg/MJ)	13	0
*n*-3 (g/MJ)	1.38	0.10
*n*-3:*n*:6	1:1	1:10
DHA + EPA (g/MJ)	1.03	0.04
GLA (g/MJ)	0.050	0.0001
Vit A (IU/MJ)	1001	888
Vit D (IU/MJ)	51	89
Vit E (IE/MJ)	30.3	29.6
Cu (mg/MJ)	0.57	0.0001
Na (g/MJ)	0.12	0.15
Mg (g/MJ)	0.04	0.0001
Taurine (mg/MJ)	138	0
Crude fat (g/MJ)	10	9
Crude fibre (g/MJ)	0.9	1.5
Crude ash (g/MJ)	3	4
Carbohydrates, NFE (g/MJ)	22	22

Abbreviations: Ca—calcium, P—phosphorus, Zn—zinc, *n*-3—polyunsaturated omega-3 fatty acid, *n*-6—polyunsaturated omega-6 fatty acid, DHA—docosahexaenoic acid, EPA—eicosapentaenoic acid, GLA—gamma-linolenic acid, Vit—vitamin, Cu—cooper, Na—sodium, Mg—magnesium, g—gramm, mg—miligramm, kJ—kilojoule, MJ—megajoule.

**Table 3:** Results of hematological variables at individual time points in dogs fed a prescription EPA + DHA rich diet and dogs fed a maintenance diet. Data are presented as mean ± standard deviation.

Parameter	EPA + DHA Rich Prescription Diet *n* = 10			Maintenance Diet *n* = 10		
	Point 0	Point 1	Point 2	Point 0	Point 1	Point 2
WBC (G/L)	9.85 ± 3.2	8.7 ± 2.1	10.7 ± 6.1	8.7 ± 2.1	7.7 ± 1.6	6.6 ± 0.1
RBC (T/L)	6.8 ± 0.6	7.4 ± 0.7	7.1 ± 0.8	7.3 ± 0.4	7.4 ± 0.4	7.9 ± 1.6
HGB (mmol/L)	10.8 ± 2.4	14 ± 3.6	9.9 ± 1.2	11 ± 1.2	14.2 ± 2.5	11.45 ± 1.5
HT (L/L)	0.47 ± 0.04	0.5 ± 0.05	0.5 ± 0.06	0.5 ± 0.06	0.5 ± 0.07	0.6 ± 0.08
MCV (fL)	70 ± 2.7	67 ± 3.2	71 ± 3.8	69 ± 4.2	67 ± 7	73 ± 5
MCH mmol/L	3.6.6.76	6.7 ± 9.1	4.4 ± 0.7	1.4 ± 0.09	1.9 ± 0.27	1.5 ± 0.1
MCHC	23 ± 6	22 ± 4	20 ± 0.1	20 ± 0.3	20 ± 0,7	20 ± 0.2
PLT (G/L)	436 ± 219	393 ± 158	386 ± 178	307 ± 180	249 ± 141	334 ± 76

WBC: white blood cell count; RBC: red blood cell count; HGB: concentration of hemoglobin; HT: hematocrit; MCV: mean red cell volume; MCH: mean mass of hemoglobin in the red cell; MCHC: concentration of hemoglobin in the red cell; PLT: platelet (thrombocytes) count.

**Table 4:** Biochemical variables at individual time points in dogs fed the EPA + DHA rich prescription diet and maintenance diet. Data are presented as mean ± standard deviation.

Parameter	EPA + DHA Rich Prescription Diet *n* = 10			Maintenance Diet *n* = 10		
	Point 0	Point 1	Point 2	Point 0	Point 1	Point 2
Na (mmol/L)	143 ± 2	141 ± 1.6	145 ± 1.1	145 ± 2.5	143 ± 1.9	143 ± 2.9
K (mmol/L)	4.3 ± 0.2	4.25 ± 0.6	4.8 ± 0.3	4.5 ± 0.04	4.5 ± 0.4	4.76 ± 0.6
Cl (mmol/L)	108 ± 2.8	110 ± 5	110 ± 0.9	109 ± 2.7	111 ± 1	106 ± 4.2
Ca^+2^ (mmol/L)	1.25 ± 0.1	1.2 ± 0.17	1.21 ± 0.08	1.3 ± 0.1	1.3 ± 0.1	1.5 ± 0.1
Mg (mmol/L)	0.7 ± 0.2	0.6 ± 0.2	0.8 ± 0.08	0.8 ± 0.04	0.76 ± 0.08	0.7 ± 0.06
Fe (mmol/L)	23 ± 9	20 ± 6	22 ± 4.3	23 ± 9.3	25.8 ± 2.9	29.7 ± 5.3
Glucose (mmol/L)	5.2 ± 1	5.7 ± 0.9	5.7 ± 2	4.9 ± 0.5	5.4 ± 0.7	4.4 ± 0.8
Urea (mmol/L)	6.6 ± 2.3	8.3 ± 4.2	6.8 ± 2.1	6.3 ± 3.2	4.5 ± 0.9	7.2 ± 3.4
Creatinine (umol/L)	64 ± 24	85 ± 38	73 ± 43	58 ± 7	58 ± 9	81 ± 5
Total protein (g%)	61 ± 6.7	59 ± 6.8	43 ± 26	65 ± 2.4	64 ± 3.7	61 ± 2.83
albumin	30 ± 4.1	31 ± 4.9	29 ± 6.6	31 ± 1.3	33 ± 0.6	34 ± 2
AspAT (U/L)	40 ± 37	39 ± 16	37 ± 5	33 ± 22	24 ± 13	23 ± 5.7
ALT (U/L)	70 ± 43	130 ± 128	64 ± 17.7	124 ± 130	67 ± 83	79 ± 94
CRP	3.1 ± 1.1	2.7 ± 0.1	1.7 ± 0.06	2.6 ± 0.9	2.6 ± 0.3	1.8 ± 0.07

AspAT: aspartate transaminase; AlAT: alanine transaminase; CRP: C-reactive protein.

**Table 5:** Morphometric and ultrasonographic measurements of subcutaneous fat thickness at particular time points. Average ± standard deviation.

Parameter	EPA + DHA Rich Prescription Diet *n* = 10			Maintenance Diet *n* = 10		
	Point 0	Point 1	Point 2	Point 0	Point 1	Point 2
Fat index %	44.7 ± 7.6	45.9 ± 4.5	50.1 ± 6.2	50.7 ± 5.4	47.7 ± 7.1	49.3 ± 9.2
Fat scapula (mm)	2.1 ± 1.7	1.8 ± 0.8	2.5 ± 0.5	2.6 ± 2.1	3 ± 0.4	3.1 ± 0.5
Fat abdomen mm)	1.8 ± 1.9	1.5 ± 0.7	1.9 ± 1.6	3.1 ± 2.1	3 ± 0.6	2.7 ± 1.9
Fat lumbar (mm)	1.8 ± 1.3	1.7 ± 0.7	1.5 ± 0.4	1.6 ± 1.7	1.2 ± 0.9	1.5 ± 0.4

**Table 6:** Electro- and echocardiographic variables at individual time points in 10 dogs fed an EPA + DHA rich prescription diet and 10 dogs fed a maintenance diet. Data are presented as mean ± standard deviation. There were no arrhythmias found and no differences between both groups.

Parameter	EPA + DHA Rich Prescription Diet *n* = 10			Maintenance Diet *n*-10		
	Point 0	Point 1	Point 2	Point 0	Point 1	Point 2
HR (beat/min)	121 ± 20	130 ± 20	135 ± 29	135 ± 16	150 ± 29	114 ± 38
Arrhytmias	no	no	no	no	no	no
LA/Ao	1.7 ± 0.3	1.47 ± 0.5	1.43 ± 0.5	1.54 ± 0.3	1.48 ± 0.3	1.45 ± 0.3
LVIDd (mm)	35 ± 6	33 ± 4	36 ± 5	35 ± 7	32 ± 5	32 ± 2
LVIDs (mm)	20 ± 4	17 ± 3	20 ± 5	19 ± 5	22 ± 7	18 ± 3
SF (%)	44 ± 6	48 ± 7.4	44 ± 10	36 ± 18	38 ± 12	44 ± 13
MR (mmHg)	5.81 ± 1.1	5.55 ± 0.99	5.64 ± 1.16	4.81 ± 0.98	5.27 ± 0.79	6.19 ± 0.87

HR: heart rate; LA/Ao: left atrium to aorta diameter ratio; LVIDd: end-diastolic internal diameter of the left ventricle; LVIDs: end-systolic internal diameter of the left ventricle; SF: shortening fraction; MR: maximal flow of mitral regurgitation.

**Table 7:** List of metabolites at 2nd time point (3 months) selected by VIP model with their percentage difference (PD) and relative standard deviation (RSD).

Metabolites	PD EPA + DHA Rich Prescription Diet vs. Maintenance Diet at 2nd Time Point	RSD EPA + DHA Rich Prescription Diet *n* = 10	RSD Maintenance Diet *n*-10
L-Alanine	8.96	71.79	69.44
Pyroglutamic acid	89.79	54.65	82.22
Propylene glycol	14.27	50.79	65.93
Uric acid	−48.02	50.76	80.71
Xanthine	−55.73	86.29	57.27
L-Tryptophan	−44.49	52.63	17.26
Kynurenic acid	−27.01	65.32	50.77
Ascorbalamic acid	7.77	72.70	71.28
Glycerophosphocholine	1.02	24.92	20.16
L-Lysine	1.92	116.77	91.71
L-Asparagine	−56.40	103.38	37.15
L-Glutamine	−27.61	60.48	117.81
Ornithine	−10.88	57.69	68.57

All variables have been scaled by autoscaling. The order of samples in the data set was randomized, and the discriminant version of the partial least square regression (PLS-DA) with the default fold procedure was implemented.

**Table 8:** List of metabolites at 3rd time point (6 months) selected by VIP model with their percentage difference and relative standard deviation.

Metabolites	PD EPA + DHA Rich Prescription Diet vs. Maintenance Diet at 3rd Time Point	RSD EPA + DHA Rich Prescription Diet *n* = 10	RSD Maintenance Diet *n*-10
L-Lactic acid	61.92	69.29	50.73
L-Alanine	−98.80	97.88	14.39
Xanthine	−115.73	24.43	58.66
Pyruvic acid	72.74	55.68	28.43
Glycerophosphocholine	−12.85	27.87	16.31
L-Asparagine	53.81	25.80	18.33

## Data Availability

Datasets are available from the authors upon request.
